# COVID-19-induced sarcopenia and physical deconditioning may require reassessment of surgical risk for patients with cancer

**DOI:** 10.1186/s12957-020-02117-x

**Published:** 2021-01-11

**Authors:** Patrick Casey, Yeng Ang, Javed Sultan

**Affiliations:** Department of Oeosphagogastric Surgery, Salford Royal Foundation Trust, Salford, UK

**Keywords:** COVID-19, SARS-CoV-2, Functional deconditioning, Sarcopenia, Frailty, Prehabilitation

## Abstract

**Background:**

The long-term physiological consequences of SARS-CoV-2 (severe acute respiratory syndrome coronavirus) infection are not known. The ability of COVID-19 to cause chronic illness, sarcopenia, and physical deconditioning may be underestimated and go beyond the anticipated respiratory sequelae. Myalgia, lethargy, and anorexia are common symptoms even in mild to moderate cases and have the potential to exacerbate frailty. How this impacts on risk-stratification for patients requiring surgery for time-critical conditions, such as malignancy, requires further urgent investigation.

**Main body:**

The deleterious effect of sarcopenia and poor physical capacity are well recognised in cancer surgery. This review commentary highlights current evidence which suggests skeletal muscle as an under recognised cause of COVID-19-related functional deconditioning. The mechanisms behind this are via direct (viral induced myositis, nutritional decline, cytokine-mediated myopathy) and indirect mechanisms (social isolation, inactivity, and psychological consequences).

**Conclusion:**

Further mechanistic research is required to explore the processes behind the deconditioning effects of SARS-CoV-2 infection and how this impacts on treatment of malignant disease.

## Main text

High-quality clinical trials are making great strides in the treatment of acute SARS-CoV-2 infection whilst the research world adjusts its focus on long-term consequences of the disease. The term ‘long COVID’ is gaining popularity in the literature and denotes a multisystem deconditioning with symptoms lasting weeks if not months. Surgical teams are encountering increasing numbers of patients who have survived COVID-19. Whilst most patients will fully recover from the illness, some will be left with lasting symptoms and the impact on perioperative risk is currently unknown. Large observational studies have shown myalgia and general weakness is seen in up to 50% of patients which can last for several months [1–6]. Currently, it is unclear why some patients take longer to recover than others but this suggests the ability of SARS-CoV-2 to cause chronic illness and physical deconditioning may go beyond the anticipated respiratory sequelae. The impact of sarcopenia and poor physical function is well described in patients requiring cancer surgery resulting in increased mortality and morbidity [7]. Assessment tools to assess the degree of COVID-related deconditioning and aid risk stratification for cancer surgery are required, especially in vulnerable patients with comorbidity and cachexia. This seems increasingly challenging given that conventional pre-operative assessment tools such as cardiopulmonary exercise testing (CPET) and spirometry are discouraged due to their aerosol risk.

Anorexia, anosmia, and weight loss are also common features of SARS-CoV-2 infection and may exacerbate nutritional deficits and muscle wasting already seen in patients with active malignancy. Some patients may never recover to their baseline functional capacity [8–10]. It is clear that those severely affected by COVID-19 with prolonged convalescence should have cancer surgery delayed where possible. Multimodal prehabilitation and rehabilitation may help to avoid lasting morbidity and reduce perioperative risk [11]. Data is lacking, however, on the deconditioning effects for patients with mild or moderate disease who are treated in the community. Any mild illness with even short periods of inactivity could see skeletal muscle loss of 0.5–6% per day [12]. Patients operated on with asymptomatic COVID-19 had increased risk of perioperative morbidity and mortality. Doglietto et al. showed that 30-day risk of mortality was significantly higher 19.5% vs 2.4%; OR 9.5, CI 1.8–96.5) compared to patient without COVID-19 [13]. As surgeons and oncologists across the world adjust pathways to safely offer treatment for malignant disease, the results from the GlobalSurg-CovidSurg Week study are eagerly anticipated. This international collaborative observational study aims to determine the optimal timing for surgery following SARS-CoV-2 infection. Early signals from around 150 patients show that pre-operative diagnosis of SARS-CoV-2 is associated with poorer peri-operative outcomes even in those who were diagnosed several weeks prior to surgery [14]. The underlying reasons for this remain unclear but could be related to sarcopenia and physical deconditioning following COVID-19. The results of their study will better inform surgical decision-making and identify patient groups that will benefit from multimodal rehabilitation (or prehabilitation).

### Skeletal muscle as a potential target

Both muscle weakness and myalgia are well recognised in COVID-19 [15]. Studies of hospitalised patients have described biochemical evidence of muscle damage [16]. Several reports of severe rhabdomyolysis, even in the absence of respiratory symptoms, suggests skeletal muscle tissue is not immune from the disease process [17–19]. Virus-induced myositis is seen with other pathogens including SARS-CoV-1 and influenza but is unclear whether immune-mediated injury due to myotoxic cytokines (such as CXCL-10, IFN-ϒ, IL-1β, IL-6, IL-17, and TNFα) or if direct viral infiltration is the predominant pathological process. SARS-CoV-2 viral particles have been detected by electron microscopy within skeletal muscle and cardiac muscle fibres whilst distressing case reports of fatal cardiac failure in children due to fulminant cardiomyopathy suggests the direct impact on muscle tissue can be profound [20]. SARS-CoV-2 enters host cells via the angiotensin-converting enzyme 2 (ACE2) receptor. Both skeletal and cardiac muscle exhibit robust expression on the ACE2 receptor on their surface as do many other tissues offering a potential viral route for direct tissue damage [21, 22]. Clinical parallels can be drawn from the SARS-CoV-1 pandemic in 2003 owing to its genetic and clinicopathological homology with SARS-CoV-2. Extensive myalgia and muscle dysfunction were reported in SARS patients. Widespread muscle fibre atrophy, fibre necrosis, myofibril disarray, and Z-disc streaming was demonstrated in post-mortem muscle tissue [23]. Furthermore, relatively fit SARS survivors were shown to have objective reductions in hand grip strength (32%) and 6-min walk test (13%) when followed up in clinic [24]. Similar functional outcomes measures in survivors of COVID-19 have also been described with measurable reductions in indices of muscle strength and physical function [25]. How long these impairment last for is unknown.

The indirect ability of the viral pandemic to cause physical deconditioning should also be considered. Some experts predict a pandemic-related increase in surgical morbidity and mortality due to social isolation and reduced activity levels in patients, where operations have been rescheduled or postponed [26, 27]. This deleterious effect will be more striking in the elderly or medically frail who also suffer psychological consequences and demotivation. For patients awaiting major cancer surgery, many community prehabilitation programs and local gymnasiums have closed evoking a rapid response to establish at-home exercise programs and telemedicine to minimise the effects of social isolation and pre-operative inactivity.

**Fig. 1:**
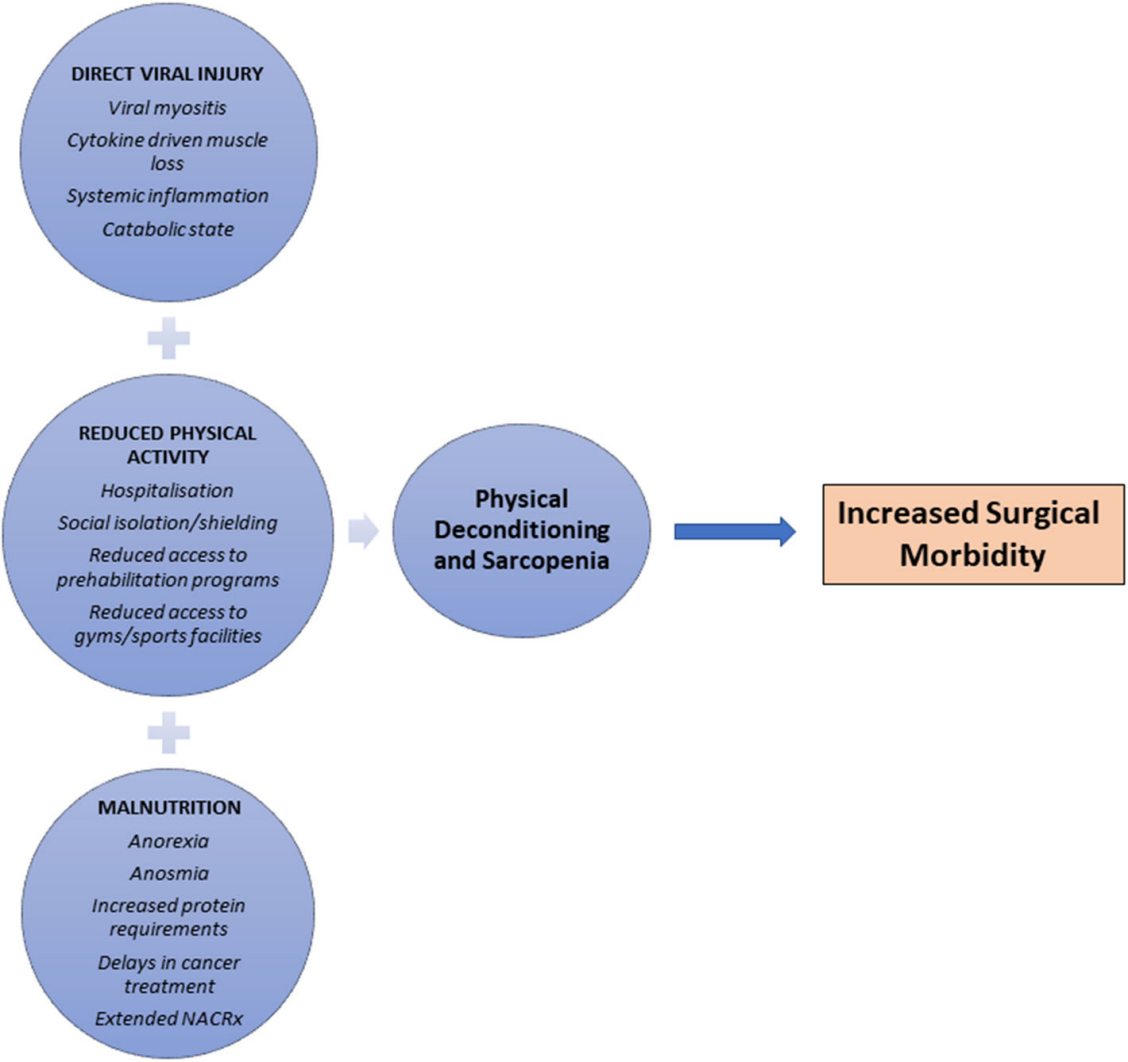
Schematic of proposed mechanisms causing deterioration in physical function and sarcopenia in patients requiring cancer surgery. Multimodal prehabilitation for COVID survivors may be required to overcome these factors. NACRx, neoadjuvant chemoradiotherapy

In conclusion, the ability of SARS-CoV-19 to cause physical decline is multifactorial and may be related to periods of convalescence, reduced appetite, chronic cardiorespiratory symptoms, social isolation, and reduced access to physical activity (Fig. 1). The direct and indirect impact on muscle strength, sarcopenia, and frailty may be underestimated and could, in part, explain the poor surgical outcomes seen in patients with previous SARS-CoV-2 infection. Functional outcome scales such as those proposed by Klok et al. may compliment standard assessment tools such as WHO performance status in assessing global functional recovery from the illness but remain unvalidated [28]. Pragmatic approaches to keep physically active prior to cancer surgery, such as home-based exercise programs, will become increasingly important as well as novel rehabilitation techniques for those worse effected by COVID-19 [29]. Going forward, we need a better understanding on how, and to what degree, COVID-19 causes physical deconditioning in mild, moderate, and severe cases alike. Whether this adjusts peri-operative risk for patients requiring cancer surgery requires further investigation to identify best timing for surgery and to tailor focussed prehabilitation for COVID-19 survivors.

## Data Availability

All data and materials used in the production of this work will be available on request.
